# Supplementary figures and images for: The Trypanosoma brucei subpellicular microtubule array is organized into functionally discrete subdomains defined by microtubule associated proteins
Source: PLoS Pathog. 2021 May 19;17(5):e1009588. doi: 10.1371/journal.ppat.1009588 (PMC8168904; doi:10.1371/journal.ppat.1009588)

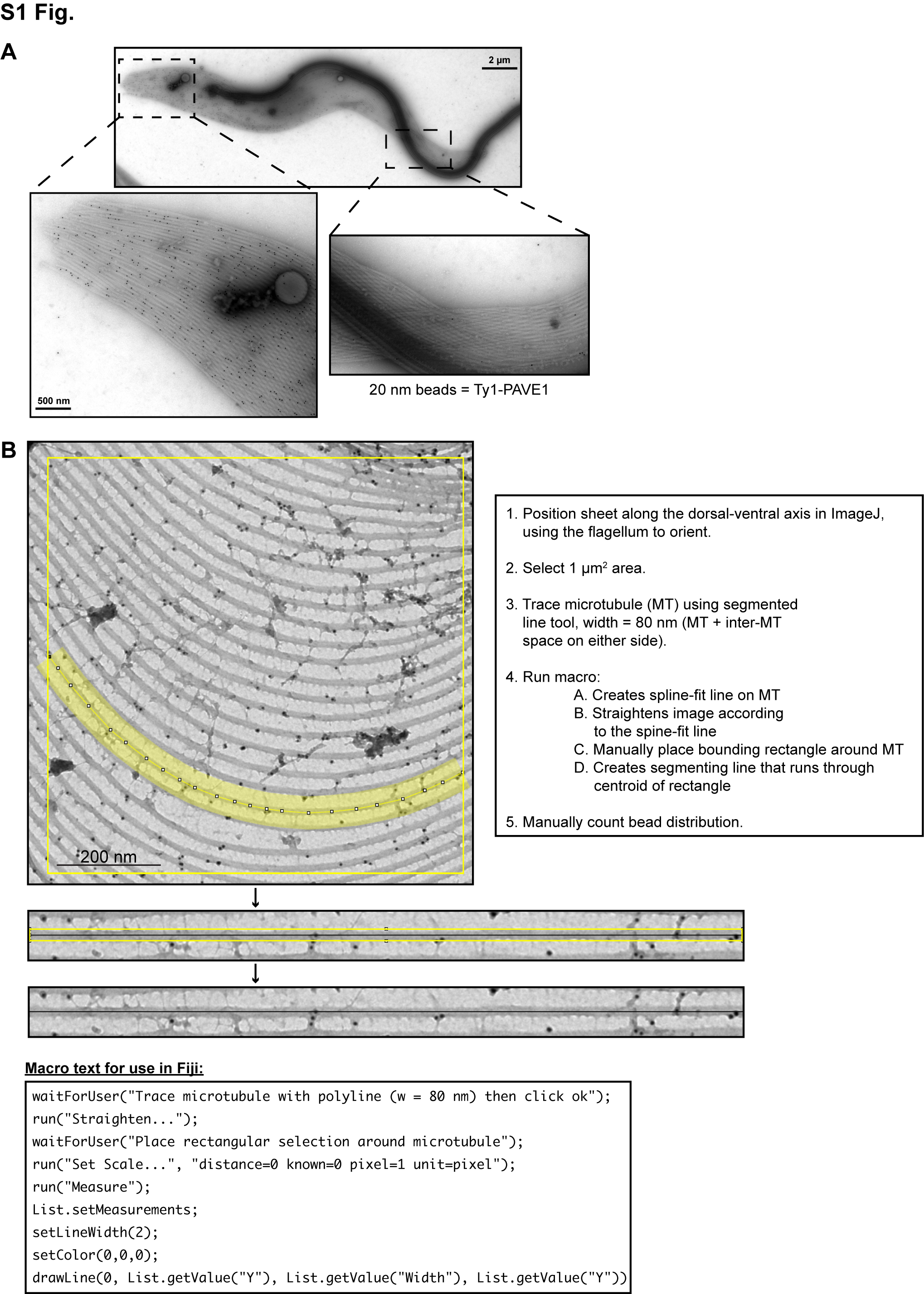

Supplement: S1 Fig — (A) A whole-mount extracted cytoskeleton immunogold-labeled for Ty1-PAVE1 with 20 nm beads. (B) A semi-automated macro was written in ImageJ to facilitate the classification gold bead distribution as dorsal, centroid, or ventral, as well as cross-link associated, along individual microtubules of subpellicular array sheets. (TIF) [file ppat.1009588.s001.tif]

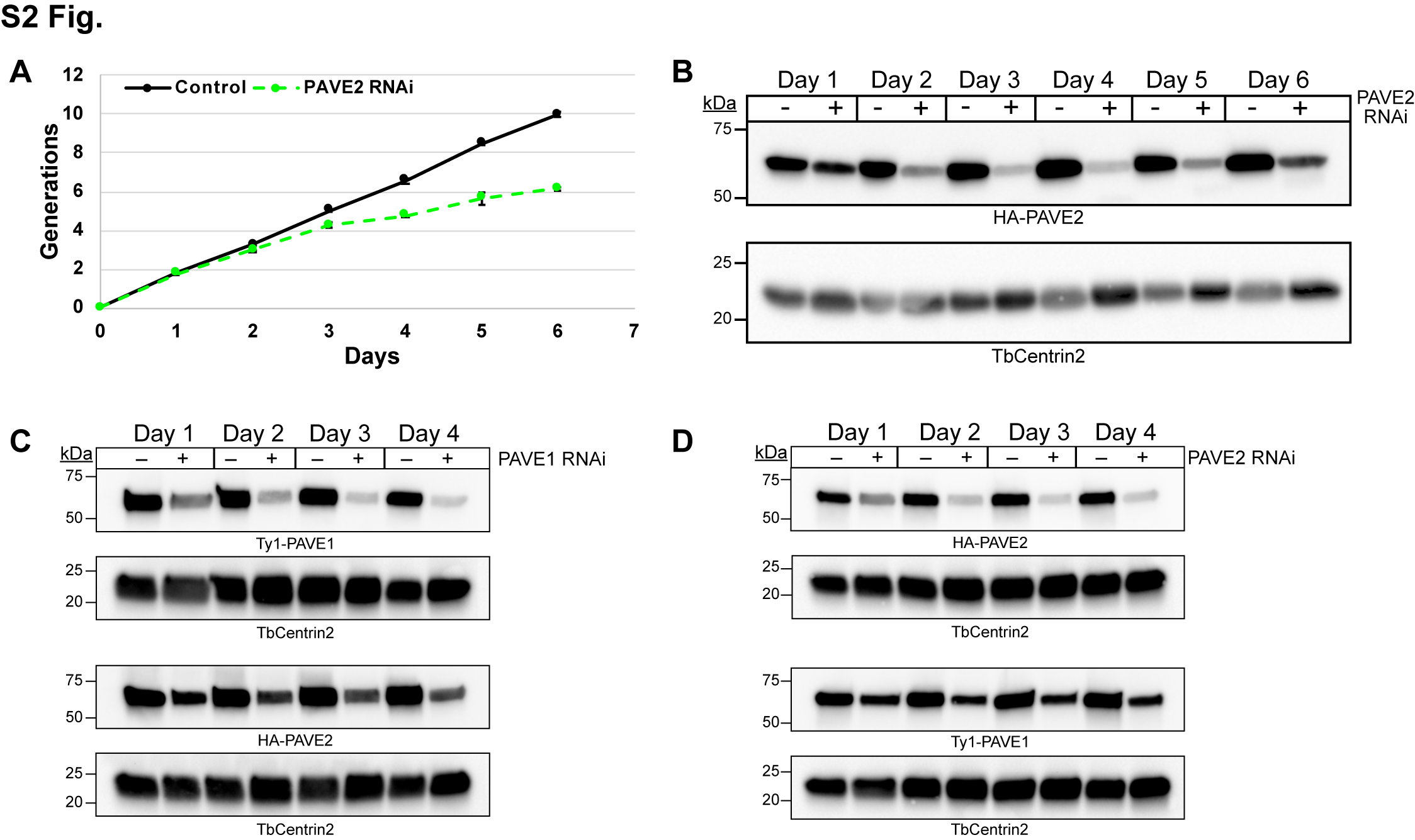

Supplement: S2 Fig — (A) Generation plot of cell growth after induction of PAVE2 RNAi. The cell density was monitored every 24 h in control and PAVE2 RNAi conditions. The curve is the mean of N = 3 independent biological replicates. (B) Lysates were collected every 24 h from control and PAVE2 RNAi cells during 6 d of PAVE2 depletion. Lysates were western blotted with antibodies against HA and TbCentrin2 as a loading control. (C) Lysates from control and PAVE1 RNAi cells in which PAVE2 is endogenously tagged were collected for western blotting every 24 h during PAVE1 depletion. Duplicate lysates were western blotted, as PAVE1 and PAVE2 are the same molecular weight. (D) Lysates from control and PAVE2 RNAi cells in which PAVE1 is endogenously tagged were collected for western blotting every 24 h during PAVE2 depletion. The lysates were blotted in duplicates as in (C). (TIF) [file ppat.1009588.s002.tif]

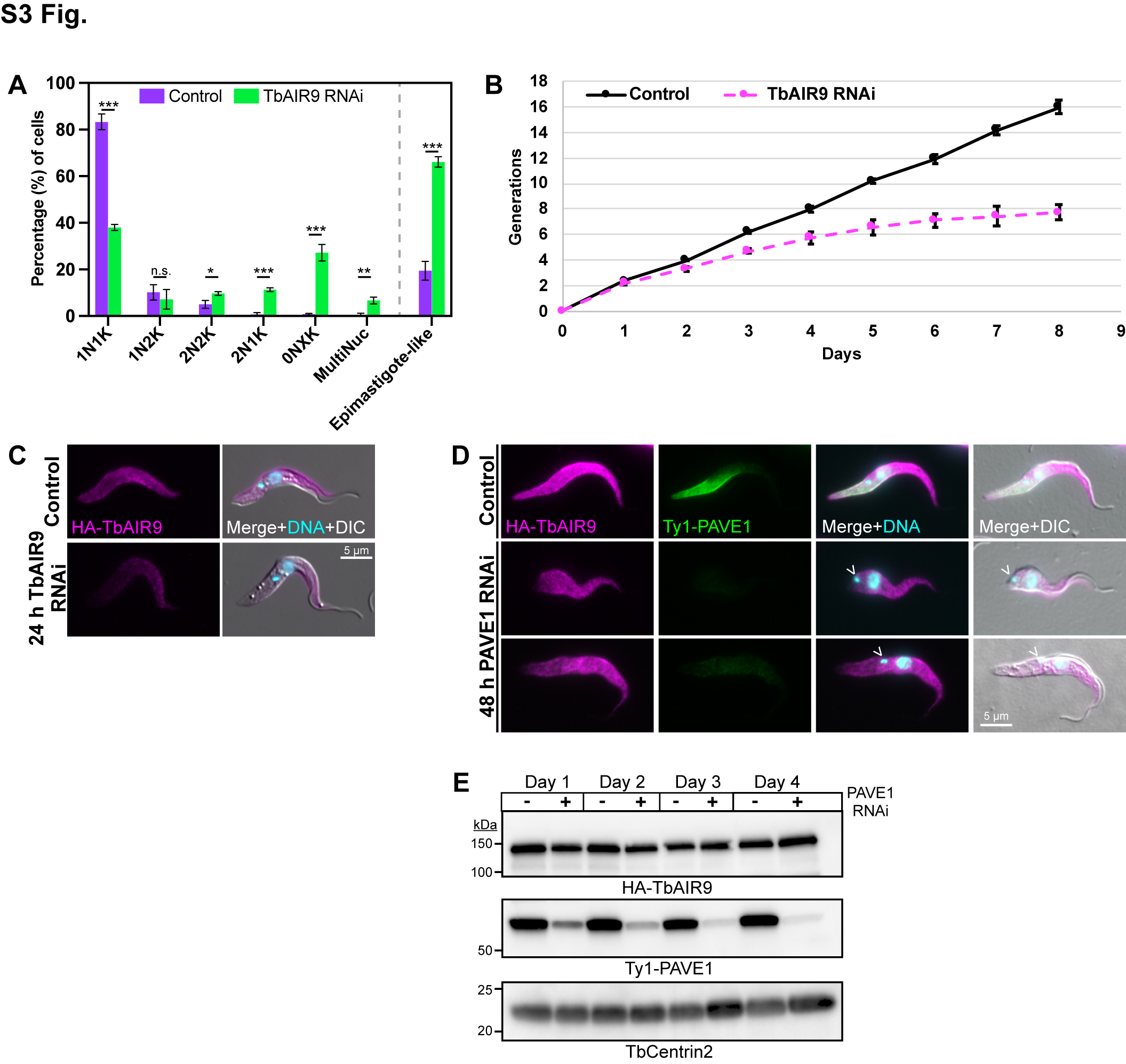

Supplement: S3 Fig — (A) Quantification of DNA states of paraformaldehyde-fixed cells after 2 d of TbAIR9 RNAi. 0NXK; anucleate cells. MultiNuc; multinucleated cells. Epimastigote-like cells were cells in which the kinetoplast was located at the midline and anterior to the nucleus; 0NXK and MultiNuc were excluded from this analysis. 200 cells were counted from N = 3 independent biological replicates. *P<0.05, **P<0.005, ***P<0.001. P-values are calculated from unpaired two-tailed Student’s t-tests using the average from N = 3 independent biological replicates. (B) Generation plot of cell growth after induction of TbAIR9 RNAi. Cell concentration was monitored in control and TbAIR9 RNAi cells every 24 h. The curve is the mean of N = 3 independent biological replicates. (C) Control and TbAIR9 RNAi cells were fixed with paraformaldehyde and stained after 24 h of TbAIR9 depletion. TbAIR9 localization initially disappears from the cell posterior during TbAIR9 RNAi, as previously reported. (D) PAVE1 RNAi was induced in cells in which TbAIR9 is endogenously tagged with the HA epitope tag. Cells were collected for fixation in paraformaldehyde after 2 d of PAVE1 depletion and stained for epifluorescence microscopy. The localization pattern of TbAIR9 does not change after PAVE1 depletion. (E) Lysates were collected from control and PAVE1 RNAi cells in which TbAIR9 is endogenously tagged every 24 h during PAVE1 depletion and western blotted. TbAIR9 stability is not affected by PAVE1 depletion. (TIF) [file ppat.1009588.s003.tif]

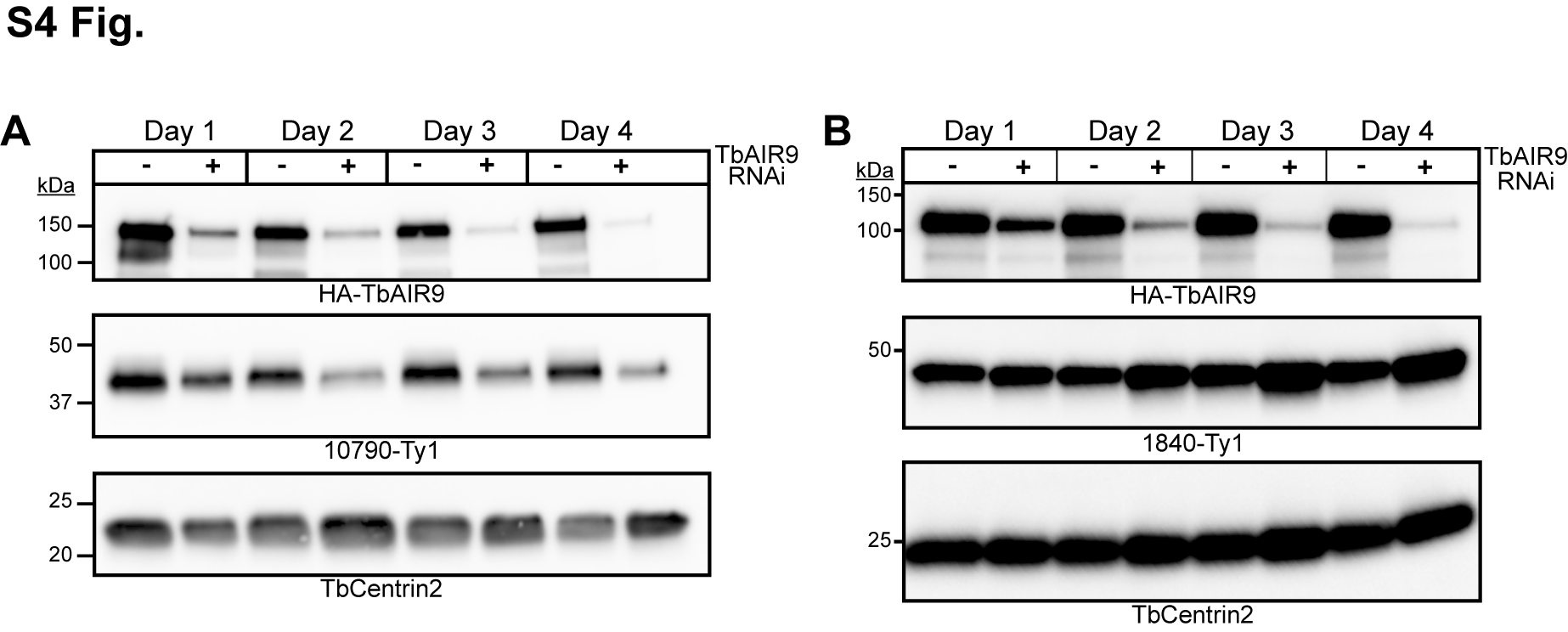

Supplement: S4 Fig — (A) Lysates were collected from control and TbAIR9 RNAi cells in which Tb927.9.10790 was endogenously tagged every 24 h during TbAIR9 depletion and western blotted. 10790-Ty1 protein levels decrease during TbAIR9 depletion. (B) Lysates were collected from control and TbAIR9 RNAi cells in which Tb927.11.1840 was endogenously tagged every 24 h during TbAIR9 depletion and western blotted. 1840-Ty1 protein levels are stable and increase during TbAIR9 depletion. (TIF) [file ppat.1009588.s004.tif]
